# Opening the door: inviting youth and parent perspectives on youth mental health emergency department use

**DOI:** 10.1186/s40900-020-00204-7

**Published:** 2020-05-27

**Authors:** Leslie Anne Campbell, David Lovas, Ellen Withers, Kylie Peacock

**Affiliations:** 1 Dalhousie University Department of Community Health and Epidemiology, Halifax, Nova Scotia Canada; 2 Dalhousie University School of Nursing, Halifax, Nova Scotia Canada; 3 Department of Psychiatry, IWK Health Centre, Halifax, Nova Scotia Canada; 4 Dalhousie University Department of Psychiatry, Halifax, Nova Scotia Canada; 5 Dalhousie University School of Occupational Therapy, Halifax, Nova Scotia Canada

**Keywords:** Patient engagement, Youth mental health, Emergency department use

## Abstract

**Background:**

Canadian Emergency Departments (EDs) have seen increasing use by children and youth for mental health concerns in recent years. This trend is likely a result of several complex factors, and researcher-posed potential explanations include gaps or access problems in primary care or mental health services, increasing prevalence or awareness of mental disorders and fear of potentially harmful outcomes, or expectations of need for urgent specialist care. Youth, parent, or caregiver perceptions and reasons for increasing mental health ED use may differ, and to date have been underrepresented in informing research directions. We sought to engage with youth and parents or caregivers served by a Canadian tertiary paediatric health centre to: 1) inform research directions for an emerging program of research in child and youth ED use for mental health care; and 2) develop strategies to support ongoing patient engagement in our research.

**Methods:**

Youth and parents were consulted to inform the development of a research engagement strategy. Partnerships with local community agencies facilitated supported engagement with both youth and parents. Group and individual in-person engagement opportunities were offered, as well as opportunities for written submissions and information sharing.

**Results:**

Youth and parents identified specific mechanisms to support engagement and for sharing ongoing opportunities and progress, including providing multiple platforms for engagement, offering separate opportunities for youth and parents or caregivers, and minimizing the potential for distress by ensuring appropriate supports and providing alternative opportunities for feedback, including written submissions. They identified lack of timely access to mental health care in primary care and community mental health settings, and accessibility, dependability, and familiarity of the ED as areas for further research.

**Conclusions:**

Strategies to mitigate potential concerns regarding distress, readiness for participation, literacy, and protection of privacy were highlighted as important considerations. Youth and parents were interested in ongoing research engagement through consultation and information sharing. Youth and parents identified areas of interest for research and refined the research team’s proposed research directions by adding contextualizing information.

**Trial registration:**

Not applicable.

## Plain English summary

### The Problem

Children and youth in Canada are going to Emergency Departments for mental health care increasingly often. Researchers have ideas about why this may be happening, including:

- Access: Not able to get timely help from family doctors or community mental health services.
- Information: More worry about the risks of mental illness.
- Expectation: Expecting better, faster care in the Emergency Department.
- Experience: Increases in mental health emergencies among children and youth.

These may be different than the reasons youth or parents seek mental health care in Emergency Departments.

### What We Did

We consulted with youth, parents, and local community youth mental health agencies to develop a research engagement plan that supported youth and parents and would help us ask the right research questions, in the right way. We consulted youth and parents separately, both in groups and individually, and made it possible to share information in person and in writing.

### What We Learned

Youth and parents identified lack of access to family physicians, gaps in mental health services, and the dependability and familiarity of the Emergency Department as important directions for research. They identified ways to engage with youth and parents that reduced possible distress and supported potential differences in participants’ confidence in communicating their opinions and reading ability. Youth were more interested in sharing their research ideas and hearing about our findings than participating on research teams.

## Background

Canadian children and youth are presenting to Emergency Departments (EDs) for mental and behavioural concerns at increasing rates. Recent data from the Canadian Institute for Health Information (CIHI) described a 75% increase in ED visit rates for mental disorders by Canadians ages 5 to 24 years between 2006 and 2007 and 2017–2018 [1]. Many (39%) children and youth presenting to EDs for mental health concerns have several repeat visits, returning to the ED three or more times, compared to 15% returning to the ED for other conditions [2]. Furthermore, this is not unique to Canada, with similar findings reported in the US [3], and Norway [4].

These trends are of concern for several reasons. First and foremost, the busy ED setting combined with the cross-sectional nature of encounters is unlikely to meet the complex mental health needs of youth and their parents or to facilitate continuity of care [5, 6]. The average length of stay in the ED for mental health visits is typically substantially longer than for other visits [7, 8]. In addition to placing pressure on the ED, patients with extended ED lengths of stay are more likely to leave before receiving care and experience dissatisfaction with health services [7]. Given the chronicity of many mental disorders, ongoing engagement with the health care system is necessary for improved patient outcomes [9].

The reasons for the increasing use of the ED for mental and behavioural crises are likely numerous and complex, and may include lack of timely access to primary care or community mental health services, perceived or real access to better or faster care through the ED, an increasing prevalence of mental disorders, growing perceptions of risk of harm or poor outcomes, or perceived need for specialized or urgent care [10, 11]. These hypotheses have been largely offered by health services researchers, clinicians, health system administrators, and policy makers. We recognize youth or parent perceptions and reasons for seeking mental health care at an ED may be different and have to date been underrepresented in health services research [12, 13]. Engagement with patients and parents in the design and implementation of research is expected to improve the relevance of studies as well as facilitate the translation of findings into clinical practice [14]. Therefore, we sought to engage with youth and parents (or caregivers) in an emerging program of research aimed at understanding increasing mental health ED presentations in a Canadian paediatric tertiary health centre (IWK Health Centre) to support the development of effective interventions.

While we did not solicit information about participants’ personal experiences, we were aware of the potential for distress in participating in discussions about ED use for mental health reasons and aimed to develop a supportive plan for engagement. Accordingly, we undertook a pre-engagement consultation process with youth and family advisory councils at the IWK Health Centre, local community youth mental health agencies, youth with lived experience, and their parents or caregivers to inform our approach.

## Methods

*Consent was not required by our Research Ethics Board as we were not conducting research. Rather, we were engaging youth and parents to collaborate in informing a program of research.*

### Pre-engagement consultation

We sought consultation from youth and parents (or caregivers) with lived experience of seeking mental health care in an ED and who were in a state of recovery to advise the development of a distress-sensitive approach to our engagement activities. Our team met with members of the IWK Youth Advisory Council and the IWK Family Leadership Council, former clients (“patients”) of IWK Mental Health and Addictions (MHA) Services and their parents, and community organizations providing peer and professional mental health supports and services to young people and their parents or caregivers.

These initial consultations aimed to identify considerations, formats, and opportunities for supporting engagement with youth and their parents or caregivers in a potentially sensitive research area, as their well being was of central importance during our development of an engagement strategy. Formats included group and individual face-to-face meetings, and telephone and emailed communication. These consultations were used to inform the development of communication materials (information materials, recruitment posters, web and social media information, and communication strategies), and to plan strategies and formats for engagement activities.

### Youth and parent or caregiver engagement

We adhered to the International Association for Public Participation (IAP2) Core Values for the Practice of Public Participation [15] for engagement activities in actively seeking participation from those likely to be affected by decisions based on our research. Our approach to writing was informed by the Guidance for Reporting Involvement of Patients and the Public (GRIPP2) [16] statement. We aimed to solicit a broad range of youth and parent or caregiver perspectives to inform our research directions, ongoing engagement, and knowledge translation strategies. As such, we used several methods of recruitment to enable representation of a range of mental health concerns, ages, sex, gender, socioeconomic backgrounds, and intensity of use of mental health supports and services including the ED. Recruitment was offered through the IWK MHA Community Liaison and advertisements in the IWK Community MHA clinics, community agencies, the research team’s website (healthyyoungminds.ca), Twitter account, and Facebook page. We did not recruit youth, parents, or caregivers in the ED, as they would likely be experiencing a mental health crisis and would be more vulnerable to any distress caused by discussing ED use.

Engagement activities reflected the learnings from our initial consultations. We offered individual and group opportunities, held separate engagement sessions for youth and parents or caregivers, and provided written information both in advance of and during sessions to help keep those participating oriented to the purpose of the discussion. Group engagement sessions were held within the community-based organizations, which provided settings familiar to participants and access to peer and staff supports. Staff from the organizations sat in each of the sessions to provide support if necessary. Food selected by youth was provided before the sessions to afford an opportunity for those participating to interact casually with the research team and break the ice. Those participating were compensated for their time by means of gift cards to local restaurants preferred by youth and of sufficient value to take a friend.

Based on our pre-engagement consultation, sessions were not recorded as this was deemed likely to inhibit sharing of opinions and would not reflect the spirit of equal collaboration (as opposed to research participation). Two research team members took field notes with the permission of those participating and ensured accurate capture of key statements. The research team debriefed following each session. Written notes were reviewed for completeness of capture of information and to identify key themes for research directions and ongoing engagement strategies.

We evaluated each engagement session with opportunities for oral and/or written feedback to accommodate varying levels of literacy and comfort in sharing feedback with the research team. Ongoing feedback was enabled through the provision of website with a communication portal, email, Facebook, and Twitter account information at the sessions and through the community partners.

## Results

### Pre-engagement consultation results

Youth and parents identified several considerations and approaches to supporting youth and parent engagement. They identified the importance of including youth, parents, and caregivers with lived experience of mental illness or seeking paediatric mental health care in an ED setting while minimizing the potential for distress. Strategies to minimize distress included holding engagement sessions in partnership with community agencies that offer peer and professional support. Youth requested that written information be provided during engagement sessions (wall posters, informational hand outs, etc.) to reinforce the objectives of the session and help those participating to maintain their focus without having to ask for clarification. Youth valued opportunities to provide written information to the research team both during in-person sessions and afterward to minimize potential distress associated with verbalizing their contributions.

For some youth or parent consultants, preparing for our conversations was the first time they had discussed their respective ED experiences with each other. They identified important differences in their reasons for seeking ED care and stressed the need to offer separate and joint (if requested) youth and parent or caregiver engagement sessions to allow for open conversations. Parents reported previously having had limited opportunities to discuss their own perspectives and valued incorporating these into research priorities.

Youth valued the inclusion of potentially marginalized groups, including those with limited literacy, unstable housing, and those who had a need for but were not currently in receipt of formal mental health and addictions services. High functioning youth (such as those enrolled in university) reflected that they were often excluded from participating in university-led research projects for course credits due to their mental illness but that in this instance were uniquely qualified to participate and suggested offering youth in-course credits where applicable for their participation. The research team collaborated with a local university to offer participation credits for research engagement.

### Youth and parent engagement results

Youth varied in sex and gender, education, socioeconomic status, literacy, and mental health care experiences. They were all within the age range of 16–25 years-old. Four in-person group engagement sessions were held in total (three youth sessions and one for parents or caregivers), hosted by either the IWK Health Centre or our community partners, with an average of 8 participants per session. One youth shared a written statement at an in-person group session. One telephone session was held with a parent living out-of-town. Youth varied in sex and gender, education, socioeconomic status, literacy, and mental health care experiences. All parents who participated were female.

#### Research directions

The following topics were highlighted as themes of research that the youth and parents we engaged with would like to see pursued and studied.

##### Access to mental health and addictions services

Youth and parents identified lack of access to mental health care in primary care or community settings and the relative accessibility of the ED as likely contributing to increasing ED use and as important areas for further research. Youth and parents described common perceptions that going to the ED was an effective means of accessing mental health services in a timely manner. Some also described specific barriers that inhibit young people’s ability to seek mental health care, including not having a family doctor, not having a health card, or not wanting to provide a health card or identification to health services.

*The emergency room is the only place to go. A 6-month wait time to see a psychiatrist is way too late. There is nothing in rural areas.*

Youth clarified that access to timely care was an important issue throughout the experience with mental illness and was not limited to its onset or during crises. Even during receipt of formal mental health services, youth turned to the ED, often more than once, for timely access to mental health professionals while they were learning to manage their symptoms.

##### Gaps in care

Gaps in communication between the ED and other parts of the mental health system (i.e. community mental health, primary care) and with parents or caregivers were consistently noted to be problematic and identified as areas for improvement. Youth and parents suggested that gaps in communication and coordination of care were likely contributing to presentations to the ED.

*It would have been helpful if the parent had an opportunity to communicate with the health care provider by writing things down. Helps the parent when the health care provider is specific.*

*We are starved for good communications in healthcare.*

##### Standards of care

There were discussions at all engagement sessions around standards of mental health care generally across health care, including primary care, the ED, and in mental health and addictions services. Youth and parents identified a need for core mental health care skills among healthcare providers because “*everyone will see mental health in their practice at some point*.” They trusted the quality of care provided at the ED, where they could access mental health and addictions clinicians.

*There has to be a basic level of knowledge and confidence (of mental health) among people working in health care.*

##### Stigma

Youth and parents perceived stigma from health care professionals toward mental health and addictions patients. Some were concerned that clinicians outside of formal mental health services were ill-equipped or not specialized enough to provide care, leading to increasing help seeking at EDs.

*You know what you’re getting when you go to the emergency room, you’re going to see someone and hopefully be taken seriously.*

##### Experience of care

While not suggested by participants as a contributor to increasing use of the ED for mental health concerns, the experience of ED care was identified as an important area for future research and service improvements. Effective communication and empathy from healthcare providers and administrative staff in the ED was identified as a pivotal issue. Both youth and parents noted that effective communication and demonstrated empathy contributed to improved patient experiences. Youth and parents stressed how youth and parents need to feel as though they are being heard, and healthcare providers should “be human” first in how they respond. Parents offered that it can be difficult to have their voice heard as a family member in a room full of clinicians.

*It’s not always about the treatment you receive, it’s about how you’re treated.*

*Socialize with them a bit. Give and receive feedback. Would be nice if people were there to make sure everything was okay.*

### Opportunities for ongoing engagement

Youth and parents identified specific mechanisms to support future engagement and for sharing ongoing opportunities and progress, including providing multiple platforms for engagement, in-person group and individual consultation, and online submissions via email, the research group’s website, and social media. With respect to social media, youth reported that while they did not use Facebook for social networking with their peers, they used the platform to learn of and participate in activities regarding mental health and addictions and expected our research group to have a presence there. Facebook pages or groups were suggested as primary avenues for connecting with parents, particularly for providing updates on the research. Online newsletters were suggested less frequently.

*You should use Facebook and YouTube. Need to use social media, lots of people are not doing it right. It is a good platform- don’t be annoying … avoid notification burnout.*

Parents noted that maintaining engagement from parents or caregivers may be difficult depending where the young person is in in their journey (i.e., diagnosis, crisis, recovery) and whether they have other children at home with mental health concerns.

Those participating were universally interested in receiving updates regarding the results of the engagement, including research directions and findings. Marginalized youth were most interested in being consulted for their input on research directions and being informed of results but were not interested in collaborating in research. They reported feeling appreciated by the opportunity to speak directly with a researcher about their perspectives and being heard.

*I think this discussion is good, but I don’t want to get emails about research you know? Like I’m glad to be a part of this and that’s cool.*

### Evaluation of engagement

*I’m glad you are asking these questions and actually listening to us*.

Youth and parents were offered the opportunity to provide either written or oral feedback to accommodate varying levels of literacy and comfort in expressing their opinions. Questions consisted of six brief “yes/no/somewhat” questions and open-ended opportunities to express what they would like to see the research team do as a result of the consultation and to provide any additional comments. Those participating consistently expressed satisfaction with the engagement, reported feeling able to voice their opinions, that their opinions were heard, and that sessions were worthwhile. Those who participated were not interested in further engagement opportunities for planning research but expressed interest in hearing about the results of ongoing and future research.

## Discussion

We engaged with youth and parents with lived experience to contribute to the development of research intended to clarify factors related to the increasing rates of paediatric presentations to EDs for mental health concerns, and to inform strategies to further engage with youth and parents or caregivers. While increasing in recent years, the perspectives of youth and parents or caregivers have often been underrepresented in the identification of research questions and the design and conduct of mental health services research studies, particularly in studies employing quantitative methodologies [12, 17].

We sought to ensure broad representation of individuals in terms of lived experience with mental illness, age, sex, gender, and intensity of use of mental health supports and services including the ED. We recognized the diversity in lived experience and intersection with socioeconomic, cultural, and other realities precluded an a priori definition of our target audience [18]. We did not refer to engagement opportunities as “patient engagement” nor define “patient”, rather allowed participants to self-identify as having direct or indirect experience with mental illness or mental health service use to avoid privileging those who successfully accessed care. We included individuals who could not or did not access formal mental health and addictions services due to stigma, geography, socioeconomic, heterosexism, or other barriers.

One of our key considerations in engaging with youth and parents was the potential for those participating to experience distress in discussing ED mental health service use, even generally. Youth and parent or caregiver wellbeing were of central importance to our research team. Individuals with mental illness have often experienced trauma or maltreatment, either prior to the development of symptoms or in the course of seeking or receiving treatment [18–21]. As such, we undertook pre-engagement consultation with youth and parents to identify and co-develop strategies to minimize potential distress and ensure support for all participating.

Youth and parents identified future areas of research in keeping with those previously identified in the literature, including problems with access to mental health services in primary care or in the community, gaps in care, or challenges providing high standards of mental health care [10, 11]. Importantly, participants identified several strategies to support those participating and foster open communication that were adopted by the research team in planning engagement activities, such as ensuring peer support for participants, offering separate sessions for youth and parents or caregivers, and providing a range of opportunities for contributions. Ensuring access to support if necessary was balanced with our desire to foster true collaboration through open and honest conversations. As such, research team members who actively provide clinical care in mental health and addictions services or in the ED who could provide support did not directly participate in the engagement sessions. Rather, participants suggested the team partner with local community agencies to conduct engagement sessions in neutral or familiar settings that would offer both peer and professional support during and after the sessions. The inclusion of peer supports known to those participating was deemed particularly beneficial in establishing relationships with local agencies and youth, role modeling in sessions, and supporting a sense of community belonging and empowerment to share opinions [22, 23].

Effective collaboration requires open and honest communication, mutual trust, and respect [24]. Parents voiced the importance of having independent opportunities for engagement, as their reasons for seeking ED care for a young person were often different than those perceived by the youth. Youth valued opportunities for engagement independent of parents or caregivers to foster more honest communication with the research team. The team was also encouraged to offer opportunities for written communication, both during engagement sessions to clarify their purpose and following sessions to allow contributions from individuals unable to attend in person or uncomfortable contributing verbally.

Engaging with patients in research aims to improve patient and health system outcomes, with the expectation that higher levels of engagement will lead to better research and better outcomes. The youth and parents with whom we consulted voiced preferences for lower levels of engagement, preferring to be consulted when considering research directions and informed of findings [15]. This may be due to barriers to ongoing or greater engagement [25–27] or reflective of their values and preferences. Attention to the training of researchers or the public, appropriate funding, and consideration of the attitudes and perceptions of researchers as means to support patient engagement in research would benefit from consideration of the values and preferences of patient participants. People who are willing or able to engage at the highest levels likely do not represent the experiences or priorities of all. Providing accessible, ongoing opportunities for lower level engagement activities may ensure a wider range of perspectives.

### Limitations

The opinions of the individuals who participated in our research engagement activities, including lack of interest in co-development of research directions or studies likely do not reflect those with different experiences or realities, or in other settings, communities, or cultures.

## Conclusions

Youth with lived experience of mental illness and their parents offered research directions to understand increased mental health ED use including lack of access to a family physician, gaps in mental health services and communication between providers and with parents, and the dependability and familiarity of the ED. Youth and parents both valued strategies that afford opportunities for youth and parents to participate separately, that mitigate potential distress by ensuring the presence of peer or other supports and that offer opportunities to engage other than verbally such as through written submissions, that accommodate varying levels of confidence or literacy, and that ensure readiness for participation and the protection of privacy. Interest in ongoing research engagement among youth and parents was limited largely to consultation and information sharing.

## Supplementary information

**Additional file 1.** GRIPP2 Short Form checklist.

## Data Availability

Not applicable.

## Abbreviations

ED: Emergency Department
IWK: IWK Health Centre
