# Supplementary material for: Opening the door: inviting youth and parent perspectives on youth mental health emergency department use
Source: Res Involv Engagem. 2020 May 27;6:26. doi: 10.1186/s40900-020-00204-7 (PMC7251901; doi:10.1186/s40900-020-00204-7)
Supplement: Supplementary file 1 — Additional file 1. GRIPP2 Short Form checklist. [file 40900_2020_204_MOESM1_ESM.docx]

| **Section/Topic** | **Item** | **Page Number** |
| --- | --- | --- |
| 1: Aim | Report the aim of PPI in the study | 7 |
| 2: Methods | Provide a clear description of the methods used for PPI in the study | 7-9 |
| 3: Study results | Outcomes—Report the results of PPI in the study, including both positive and negative outcomes | 9-15 |
| 4: Discussion and conclusions | Outcomes—Comment on the extent to which PPI influenced the study overall. Describe positive and negative effects | 15-17 |
| 5: Reflections/critical perspective | Comment critically on the study, reflecting on the things that went well and those that did not, so others can learn from this experience | 17-18 |

**GRIPP2 Short Form – Opening the Door**
